# Absorbance biosensors-based hybrid $\mathbf {MoS_{2}}$ nanosheets for *Escherichia coli* detection

**DOI:** 10.1038/s41598-023-37395-4

**Published:** 2023-06-23

**Authors:** Son Hai Nguyen, Phan Kim Thi Vu, Mai Thi Tran

**Affiliations:** 1 School of Mechanical Engineering, Hanoi University of Science and Technology, Hanoi, 100000 Vietnam; 2 College of Engineering and Computer Science, VinUniversity, Hanoi, 100000 Vietnam; 3 VinUni-Illinois Smart Health Center, VinUniversity, Hanoi, 100000 Vietnam

**Keywords:** Biochemistry, Biological techniques, Biophysics, Biotechnology, Biomarkers, Engineering, Materials science, Nanoscience and technology, Physics

## Abstract

Detecting *Escherichia coli* is essential in biomedical, environmental, and food safety applications. In this paper, we have developed a simple, rapid, sensitive, and selective *E. coli* DNA sensor based on the novel hybrid-type $\text {MoS}_\text {2}$ and $\mathrm {(NH_4)_6Mo_7O_{24}}$ nanosheets. The sensor uses the absorbance measurement to distinguish among the DNA of *E. coli*, *Vibrio proteolyticus*, and *Bacillus subtilis* when implemented in conjunction with $\text {NH}_\text {2}$-probes. Our experiments showed that the absorbance increased when sensors detected *E. coli* DNA, whereas it decreased when sensors detected *V. proteolyticus* and *B. subtilis* DNA. To the best of authors’ knowledge, there are no reports using the novel hybrid-$\mathrm {MoS_2}$ and $\mathrm {(NH_4)_6Mo_7O_{24}}$ materials for differentiating three types of DNA using cost-effective and rapid absorbance measurements. In addition, the label-free *E. coli* DNA biosensor exhibited a linear response in the range of 0 fM to 11.65 fM with a limit of detection of 2 fM. The effect of $\text {NH}_\text {2}$-probes on our sensors’ working performance is also investigated. Our results will facilitate further research in pathogen detection applications, which have not been fully developed yet.

## Introduction

The gram-negative bacterium *Escherichia coli* causes enteritis, blood sugar infections, urinary tract infections, and meningitis in infants^1^, as well as deafness, blindness, and death^2^. Prompt and effective detection of *E. coli* is necessary to save lives^2,3^. To detect *E. coli*, researchers usually extract them into double-stranded DNA (ds-DNA) or single-stranded DNA (ss-DNA)^4^, then use the techniques such as polymerase chain reaction (PCR) and enzyme-linked immunosorbent assay (ELISA) for DNA detection. However, these methods are time-consuming and costly and cannot be used for on-site diagnosis^5,6^. Hence, a biosensor based on nanomaterials, a new point-of-care method that detects pathogens with high selectivity and sensitivity, is attracted huge attention recently^7^. Some biosensing technologies have been developed, such as electrochemistry^8,9^, colorimetry^10,11^, and field effect transistors^12^. Among these techniques, optical biosensors based on nanomaterials are commonly used to detect DNA due to their abilities in real-time monitoring of measuring the DNA with high sensitivity, selectivity, and multi-analyte detection^13^. It has also been reported that the probe can enhance the sensitivity and selectivity to detect targeted DNA^12,14^. Despite these numerous advantages, it’s important to consider potential challenges associated with optical biosensors based on nanomaterials, including potential toxicity of some nanomaterials, the need for careful control over nanomaterial synthesis and modification, and challenges related to the scale-up and commercialization of these technologies.

Pure Molybdenum disulphide ($\mathrm {MoS_2}$) nanosheets is a 2D transition metal dichalcogenide, a typical graphene-like material^15–18^. They show strong adsorption ability for single-stranded DNA (ssDNA)^8,10,12,19–22^. Apart from the properties, $\mathrm {MoS_2}$ also gains attention based on its easy-to-find materials and uncomplicated protocol^23^. Moreover, $\mathrm {MoS_2}$ has been observed to have biological compatibility with human bodies in its applications, such as curing cancer and Alzheimer’s^24^. Recently, the nanostructured hybrid of $\mathrm {MoS_2}$ attracted much attention due to the ability to change the composition and properties of the excitation light^25^ and process ultrafast and nonlinear optical properties^26^. Hybrid $\mathrm {MoS_2}$ nanosheets have several advantages over pure $\mathrm {MoS_2}$ nanosheets, such as improved sensitivity, selectivity, and stability, and they can enable the detection of a broader range of analytes^27–29^. Furthermore, hybrid-type $\mathrm {MoS_2}$ nanosheet is better than pure semiconductor materials in safely injecting into the human body^16,20,22,30^. These hybrid nanomaterials intrigue properties and potential applications in sensing point-of-care. Therefore, using this hybrid material as a sensing material for optical biosensors is an area of our interest. This hydrid 2D structures with a robust light-mater intercalation motivated us to design new and cost-effective composite 2D materials to detect pathogenic DNA. We aim to prepare an affordable, compatible, and simple fabricated hybrid $\mathrm {MoS_2}$-3R nanosheets and then utilizing them to detect DNA based on the optical measurements.

In the next section, we prepared a new hybrid-type of $\mathrm {MoS_2}$ nanosheets by a simple and fast hydrothermal method. Their structures and morphologies were examined by X-ray diffraction (XRD) and Scanning Electron Microscope (SEM) images. Then the hybrid $\mathrm {MoS_2}$ nanosheets, in conjunction with an amine-probe, provided a rapid and sensitive sensing platform for differentiating *E. coli*, *V. proteolyticus*, and *B. subtilis* DNAs based on UV-vis spectroscopy measurement. In our experiments, the amine -probe-$\mathrm {MoS_2}$ nanosheet system is built as a biosensor. The performance of the biosensors with different $\mathrm {MoS_2}$ concentrations with *E. coli* DNA was also studied.

## Results and discussion

### The morphology and structure of the materials

**Figure 1:**
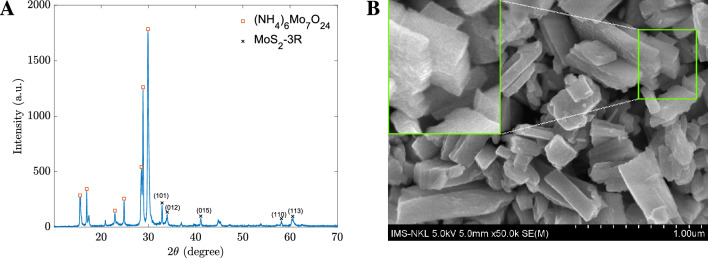
**(A)** XRD patterns and **(B)** SEM image of synthesized materials. The inset is an enlarged portion with applied contrast enhancement.

The synthesized materials’ structure, morphology, and absorbance properties were examined by XRD and SEM observations. In Fig. 1A, the composition of hybrid-type $\mathrm {MoS_2}$ includes $\mathrm {MoS_2}$-3R (card no PDF#17-0744) and $\mathrm {(NH_4)_6Mo_7O_{24}}$ (PDF#23-0784). $\mathrm {MoS_2}$-3R shows the diffraction peaks from (101), (012), (015), (110), and (113) planes, which correspond to the peaks centered at the 2$\theta$ angles of $33.03^\circ$, $34.06^\circ$, $41.11^\circ$, $58.32^\circ$, and $60.50^\circ$, respectively (PDF#17- 0744, using JADE software by MDI Materials Data). Because the hydrothermal process happened in a short period of time, 5 hours at $180\,^\circ$C; hence along with $\mathrm {MoS_2}$-3R, the precursor chemical Ammonium Heptamolybdate Tetrahydrate $\mathrm {((NH_4)_6Mo_7O_{24})}$ is still found in the resultant composite. However, based on the SEM image shown in Fig. 1B, the hybrid material clearly shows the multi-layer sheets of nanomaterials. Hence, we hypothesized that $\mathrm {(NH_4)_6Mo_7O_{24}}$ in the formation of lamellar structure $\mathrm {MoS_2}$ species in which adjacent layers are filled with $\mathrm {NH_4^+}$ ions. Therefore, it can be concluded that either $\mathrm {(NH_4)_6Mo_7O_{24}}$ functionalizes the $\mathrm {MoS_2}$ surface or $\mathrm {(NH_4)_6Mo_7O_{24}}$ molecule fragments.

### The absorbance of biosensors with different types of DNA

**Figure 2:**
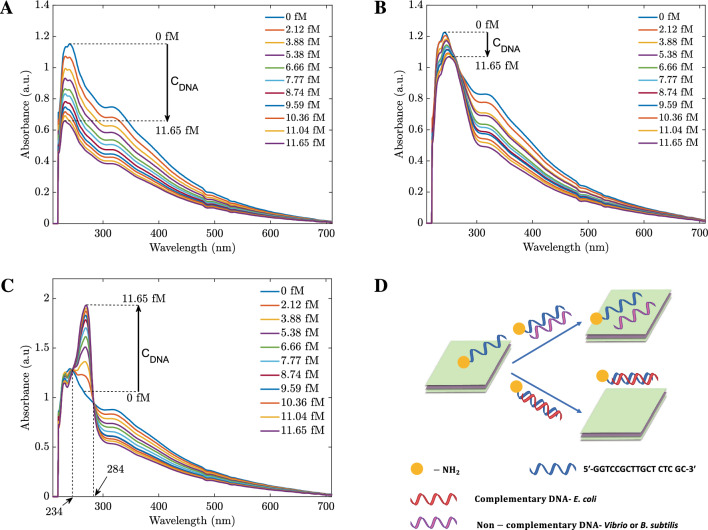
Adsorption spectra of hybridization of 0.031 mg/L hybrid $\mathrm {MoS_2}$ nanosheet solution with various concentrations of *B. subtiis* **(A)** , *V. proteolyticus* **(B)** and *E. coli* **(C)** DNAs from 0 to 11.65 fM, respectively. **(D)** The schematic of *E. coli* DNA sensing mechanism.

In this section, the absorbance properties of the hybrid-type $\mathrm {MoS_2}$ nanosheets in the range of 220–700 nm are investigated. In our experiments, the sensing materials with a concentration of 0.031 g/L hybrid $\mathrm {MoS_2}$ nanosheet solution were exposed to different concentrations of three types of DNA, *B. subtilis*, *V. proteolyticus*, and *E. coli*. It is worth noting that *V. proteolyticus* and *E. coli* are gram-negative bacteria, while *B. subtilis* is a gram-positive bacterium^31^. As shown in Fig. 2A,B, the absorbances of *B. subtilis* and *V. proteolyticus* decreased when the DNA concentration increased for the whole wavelength range from 220 to 700 nm. On the other hand, in Fig. 2C, the absorbances increased with the concentrations of *E. coli* DNA in the range of 234 nm to 284 nm wavelength. The experimental result proposes a new selective method for differentiating the three types of DNAs. In the presence of $\mathrm {NH_2}$-5′-GGTCCGCTTGCT CTC GC-3′ probe, when the excitation light is from 234 to 284 nm, *E. coli* DNA is easily detected from the others. The interpretation of the different absorption spectra might be due to the differences in the adsorption of dsDNA and ssDNA on the hybrid $\mathrm {MoS_2}$ nanosheets. It has been reported that the adsorption energy of dsDNA is much less than that of ssDNA^32^. When the different DNAs were added, only *E. coli* DNA was complementary with the probes to form dsDNA. The weak interaction of dsDNA weakens the dielectric screening from the ssDNA case, leads to a shift in absorbance peak and enhances the absorbances.

**Figure 3:**
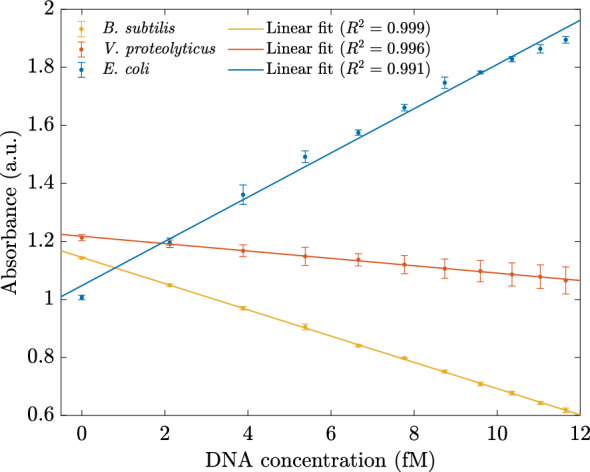
The linear relationship between DNA concentrations and the absorbance of three types of DNA in the range of 0–11.65 fM was taken at the wavelength of 255 nm. Error bars represent the standard deviations of fifteen measurements.

To further explain the detection mechanism, Fig. 2D depicts the schematic illustration of the adsorbed ssDNA on the hybrid $\mathrm {MoS_2}$ surface^33^. The ssDNA could bind to the surface of hybrid $\mathrm {MoS_2}$ and modulate the dielectric environment of $\mathrm {MoS_2}$. As the ssDNA is hybridized with its complementary DNA, the interaction between the formed dsDNA and hybrid $\mathrm {MoS_2}$ is so weak that it would be far away from the surface of $\mathrm {MoS_2}$, resulting in the dielectric environment transforming from DNA to water. Furthermore, in the combined system, the resonance absorbance of the DNA molecule and $\mathrm {MoS_2}$ nanosheets are coupled, leading to hybridized quantum molecule-classical materials^34^. Hence, the absorbance increases with the *E. coli* DNA concentrations. On the other hand, if the DNA cannot be coupled with the $\mathrm {MoS_2}$ nanosheets, when we add more DNA, the concentration of $\mathrm {MoS_2}$ is reduced and more ssDNA bind to the surface of hybrid $\mathrm {MoS_2}$; thus, the absorbance is diminished, corresponding to the experimental results of the probe and mismatched ssDNA due to the absorbance of DNA is significantly weaker than the absorbance peak of hybrid $\mathrm {MoS_2}$ nanosheets. In addition to differentiating between *E. coli*, *V. proteolyticus*, and *B. subtilis*, the absorbances of the biosensors varied linearly with the DNA concentration in the range of 0 fM to 11.65 fM in the resonant region of 234–284 nm wavelength. Figure 3 demonstrates the sensors’ performances with different DNA concentrations at 255 nm. This figure also shows that only *E. coli* has a positive slope, confirming the boosting phenomenon in absorbance. The result was obtained from the average of 15 measurements and the hybrid-type $\mathrm {MoS_2}$ nanosheet concentration was 0.031 g/L.

### Absorbance biosensor based on hybrid-type $\mathbf {MoS_2}$-3R with *E. coli*

**Figure 4:**
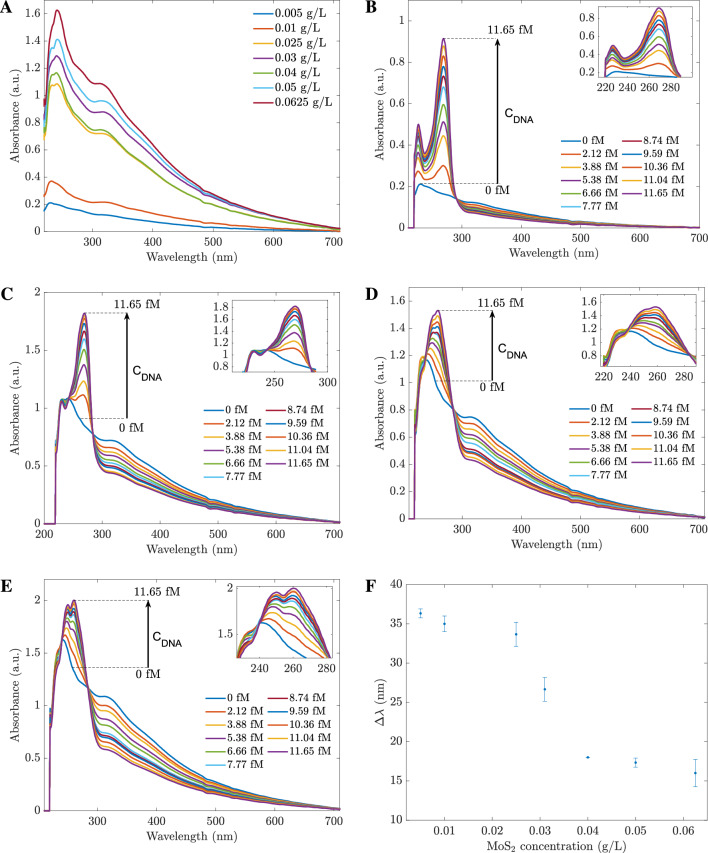
(**A**) The absorbances of the hybrid $\mathrm {MoS_2}$ based sensors with various sensing concentrations before contacting with DNA. Absorbance changes of *E. coli* DNA sensors with different hybrid-type $\mathrm {MoS_2}$ nanosheets concentrations: (**B**) 0.005 g/L, (**C**) 0.025 g/L, (**D**) 0.04 g/L, (**E**) 0.0625 g/L. (**F**) The peak shift for different types of hybrid-types $\mathrm {MoS_2}$ nanosheet sensors in contact with *E. coli* DNA. 0 fM lines are associated with the hybrid $\mathrm {MoS_2}$ nanosheet spectra before contact with DNA. The values were calculated from 6 measurements.

As demonstrated in the previous section, the complementary amine probe can be used to detect *E. coli* by the enhancing effect between 234 and 284 nm. In this section, the sensitivity of *E. coli* DNA sensors with different sensing material concentrations is studied. For quantitative analysis, the biosensors were prepared with varying hybrid-type $\mathrm {MoS_2}$ nanosheets concentration ranging from 0.005 to 0.0625 g/L and the *E. coli* DNA concentrations were from 2 to 11.65 fM. First, the absorbances of the sensors before contacting with DNA were determined and are shown in Fig. 4A. From the result, the absorbance of sensors increases as the concentration of sensing materials increases. Next, these *E. coli* DNA sensors were examined with different *E. coli* DNA concentrations. The absorbance spectra of four different hybrid-type $\mathrm {MoS_2}$ nanosheet concentrations are displayed in Fig. 4B–E. The plots indicate that the higher the sensing material concentration is, the less change in the responded absorbance.

As observed in Fig. 4B–E, the origin peaks of sensing materials are located at roughly 234 nm, even with different concentrations. After contacting with *E. coli* DNA, depending on the origin sensing concentrations, either the spectra have two peaks, or the location of a higher intensity peak changed. In addition, the locations of the highest peaks in the absorbance spectra changed when the sensor contacted *E. coli* DNA. For instance, when the sensing concentration level was as low as 0.005 g/L, the enhancing effects were observed at 234 and 270 nm peaks, and the peak shift $\Delta \lambda$ was 36 nm. The peak shift is demonstrated in Fig. 4F. The higher concentration of sensing material is, the narrower the peak shift is. This phenomenon can be explained by the fact that DNA nucleobases have a limited optical absorption, in comparison to that of hybrid-$\mathrm {MoS_2}$. Hence, with the higher sensing concentration, the induced absorbance in the presence of DNA nucleobases shows less absorbance peaks and wavelength shifting of the $\mathrm {MoS_2}$ absorbance peak^34^.

**Figure 5:**
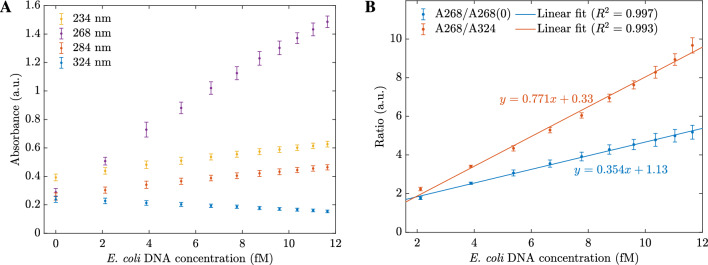
(**A**) The absorbances changed with *E. coli* DNA concentrations taken at different wavelengths; (**B**) The calibration lines of *E. coli* DNA sensors were based on two different ratios.

**Figure 6:**
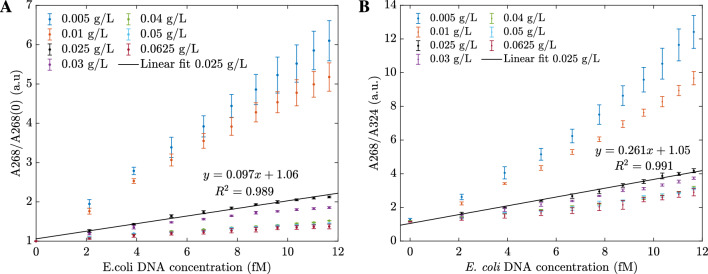
(**A**) The A268/A268(0) calibration lines for different *E. coli* DNA sensors; (**B**) The A268/A324 calibration lines for different *E. coli* DNA sensors.

The 0.01 g/L hybrid $\mathrm {MoS_2}$ sensor was examined as an example for further study. The absorbances were measured at 234 nm, 268 nm, 284 nm, and 324 nm. Here, 234 nm is the origin peak of sensing materials, 268 nm is the peak when in contact with *E. coli* DNA, 284 nm is the peak where the less change in absorbance (isosbestic point) and 324 nm is the other peak of origin material (outside the resonant bandwidth), always shows the quenching effects in response to any DNAs. The absorbances at four wavelengths are shown in Fig. 5A. With the highest slope, the enhancing effect at 268 nm is an excellent indicator to detect *E. coli*. Therefore, we introduced two quantities to use for calibration lines. The first quantity is a ratio of A268/A268(0), where A268 and A268(0) are the absorbances of sensors after and before adding *E. coli* DNA. The second quantity is a ratio of A268/A324, where A268 and A324 are the absorbances at 268 nm and 324 nm on the same absorbance spectrum. As shown in Fig. 5B, both ratios increased linearly with the concentrations of *E. coli* DNA. Since A268/A324 shows a higher slope; hence, the ratio A268/A324 can be used to determine the *E. coli* DNA concentration. The experiments were repeated for other sensors with different hybrid $\mathrm {MoS_2}$ concentrations to build the calibration lines. As shown in Fig. 6, sensors of 0.005 g/L and 0.01 g/L have the highest slopes. However, the error bars are broader than the other sensors with lower starting absorbance. Hence, the 0.025 g/L or 0.031 g/L hybrid $\mathrm {MoS_2}$ nanosheets are recommended for *E. coli* DNA sensors.

**Figure 7:**
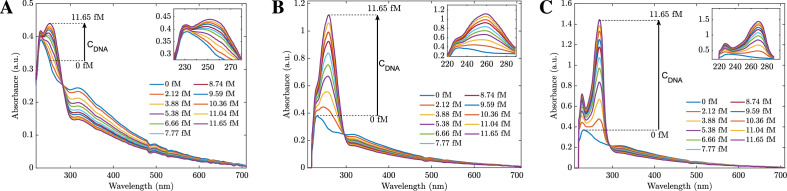
The absorbance spectrum of *E. coli* DNA sensors (**A**) without the probe, (**B**) with the probe but without $\mathrm {NH_2}$, (**C**) with $\mathrm {NH_2}$-probe. The concentration of sensors was 0.01 g/L hybrid $\mathrm {MoS_2}$ nanosheets. 0 fM lines are associated with the hybrid $\mathrm {MoS_2}$ nanosheet spectra before contact with DNA.

### Effect of an amine-probe on the absorption of 2D hybrid $\mathbf {MoS_2}$ biosensors

In the previous section, our results show that hybrid $\mathrm {MoS_2}$ nanosheets combined with amine-probe can provide a rapid and sensitive sensing platform for detecting *E. coli* DNA based on UV-vis spectroscopy measurement. The probe-amine-hybrid $\mathrm {MoS_2}$ nanosheet system is built as a sensor. Here, the probe $\mathrm {NH_2}$-5′-GGTCCGCTTGCT CTC GC-3′ is selected to detect the complementary target *E. coli* DNA according to the Watson–Crick base-pairing rules^35^. The probe was modified with amine (-$\mathrm {NH_2}$) to bond with the $\mathrm {MoS_2}$ surface and thus can enhance the absorbance. To verify our hypothesis, we repeated the experimental steps to measure UV-vis measurements with three different sensors: the first one without a probe (Fig. 7A), the second one with a probe without $\mathrm {NH_2}$ (Fig. 7B), and the third one with the amine probe (Fig. 7C). In all cases, the absorbances were enhanced. However, the shapes of the spectra and the rate of change were different. In particular, without a probe, the absorbance changes were slight (less than 0.05). When the added probe was 5′-GGTCCGCTTGCT CTC GC-3′ (without $\mathrm {NH_2}$), the first peak at 234 nm disappeared when adding DNA, and at the wavelength of 260 nm, the absorbance changed significantly (Fig. 7B). Lastly, when we used the amine-5′-GGTCCGCTTGCT CTC GC-3′, the enhancement was observed at two peaks of 234 nm and 268 nm. After adding 2 fM DNA, the second absorbance at 268 appeared, and the absorbance increase was even higher than the case in Fig. 7B.

**Figure 8:**
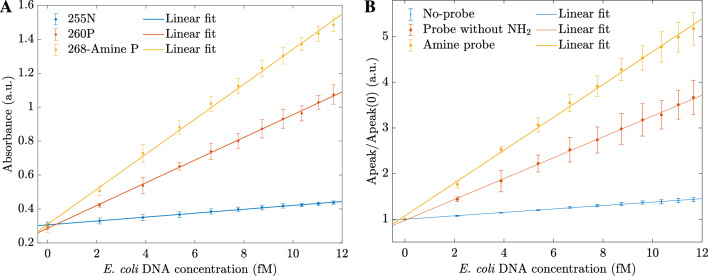
(**A**) The absorbance changes with the various concentrations of DNA of three types of sensors: 255 N denotes for absorbance at 255 nm of the sensor without the probe, 260P denotes the absorbance at 260 nm of the sensor with the probe but without $\mathrm {NH_2}$, and 268-amine P stands for the absorbance at 268 nm of the sensor with amine probe. (**B**) Comparison of three sensors without a probe, with a probe but without $\mathrm {NH_2}$, and with amine-probe using ratios at the second peaks derived from Fig. 7.

For comparison, the absorbance changes of the sensors at different peak wavelengths are plotted in Fig. 8A. The figure shows that the amine probe enhanced the sensitivity of the sensors at the resonant peak, while the slope was minimal in the case without the probe. Because for each biosensor, the enhanced absorbance peaked at different wavelengths, 255 nm, 260 nm, and 268 nm for biosensors without the probe, sensors with the probe but without $\mathrm {NH_2}$, and sensors with amine probe, respectively. Then, we plotted the graph for the introduced ratio of Apeak/Apeak(0) as shown in Fig. 8B. The amine probe boosted the sensitivity of the sensors (the slopes were more extensive) and increased the precisions. Our experiments showed that the simple configuration of amine probe-hybrid $\mathrm {MoS_2}$ nanosheets had great potential for *E. coli* DNA detection with high sensitivity and selectivity in the range of 0-11.65 fM with a limit of detection (LOD) of 2 fM, which is much lower than other sensors based on hybrid $\mathrm {MoS_2}$ nanosheets. For example, Xiang et al. reported a $\mathrm {MoS_2}$ nanosheet-based fluorescent biosensor for protein detection with a detection limit of 0.67 ng/mL^36^. Alexaki et al. reported two-dimensional dichalcogenide materials, $\mathrm {MoS_2}$ and $\mathrm {WS_2}$, with the LOD of 5 M^37^. Huang et al. reported a novel $\mathrm {MoS_2}$-based fluorescent biosensor for DNA detection via hybridization chain reactions (HCRs) with the LOD of 15 pM^38^. The applications of hybrid-$\mathrm {MoS_2}$ are still in their early stages and have not been fully explored. To the best of the authors’ knowledge, there are no existing reports on the use of the novel hybrid-$\mathrm {MoS_2}$ and $\mathrm {(NH_4)_6Mo_7O_{24}}$ materials for differentiating three types of DNA using cheap and fast UV-vis spectroscopy. Our result will facilitate other works in pathogen detection applications that are not fully developed yet..

## Conclusion

In this study, we successfully created a simple biosensor for *E. coli* DNA using hybrid $\mathrm {MoS_2}$ nanosheets and demonstrated its absorbance-enhancing ability. The absorbance of *V. proteolyticus* and *B. subtilis* DNA was quenched during the working processes. The sensor can detect DNA at 0-11.65 fM without any amplification strategy or dopant process. The biosensing platform could also detect DNA with high sensitivity and repeatability, with a detection limit of 2 fM. Hence, a homogeneous quantitative DNA analysis was provided with a short turnaround time, simple operation, and relatively high sensitivity. Furthermore, the complementary target DNA could be distinguished from mismatched DNA through the absorbance spectra of Amino-probe hybrid $\mathrm {MoS_2}$. This work could promote the research of novel sensing platforms by coupling nanomaterials with biomolecular recognition events. Therefore, the findings suggest that such a biosensor is promising for nucleic acid detection, particularly quantitative DNA methylation analysis at the point of care. The following steps will explore the sensor’s performance with photoluminescence measurements. We will also adjust the prepared conditions or dope other compositions to boost the sensitivity and selectivity more.

## Methods

### Chemicals and probe to detect *E. coli* DNA

The chemicals used in this research without further purification were Ammonium Heptamolybdate Tetrahydrate ($\mathrm {(NH_4)_6Mo_7O_{24}}$, 99.0%, Tianjin Chemical Reagent Factory, Tianjin, China), Thioacetamide ($\mathrm {C_2H_5NS}$, 99.0%, Shanghai Zhanyun Chemical Co., Ltd, Shanghai, China), Ethanol ($\mathrm {C_2H_5OH}$, 99.5%, Xilong Scientific Co., Ltd., Guangdong, China), and deionized (DI) water. The oligonucleotide probe was designed to specifically target *E. coli*, using the sequence: amine-5′-GGTCCGCTTGCT CTC GC-3′^35^. *E. coli*, *V. proteolyticus*, and *B. subtilis* DNAs were pretreated by heating at $95\,^\circ$C for 5 min and then placed in an ice bath for 1 min. All probes were purchased from PHUSA genomics Co., Ltd, Can Tho, Vietnam.

### Synthesis of hybrid-type $\mathbf {MoS_2{-}3R}$ nanosheets and absorbance measurement

Hybrid-type $\mathrm {MoS_2}$ nanosheets were prepared using the hydrothermal method^39^. The process was as follows. First, 5 g of $\mathrm {(NH_4)_6Mo_7O_{24} \cdot 4H_2O}$ and $\mathrm {C_2H_5NS}$ were completely dissolved in 20 mL of deionized water and stirred separately for 10 min. Then we mixed and stirred them for 5 min. Next, the mixture was slowly added 20 mL Ethanol and stirred for 30 min. The precipitation was transferred to an 80 mL Teflon-lined stainless-steel autoclave, kept at $180\,^\circ$C for 5 h, and then allowed to cool naturally to room temperature. Finally, the products were collected by centrifugation at 5000 rpm for 4 min, washed three times with DI water and Ethanol, and dried in a vacuum at $60\,^\circ$C for three hours. The structure and morphology of synthesized materials were characterized by Rigaku MiniFlex600 (for X-ray patterns) and HITACHI-S4800 (for SEM images).

### DNA extraction method

Three bacteria are provided by the microbiology and genetics lab at Hanoi University of Science and Technology, Hanoi, Vietnam. The chemicals were used for these extractions includes the 2% w/v CTAB (Biobasic, Canada), 100 mM Tris-HCl pH 8.0 (Biobasic, Canada), 20 mM EDTA (Biobasic, Canada) and 1.4 M NaCl (Merck, Germany). Before the sterilization process, the pH of the lysis buffer was adjusted to 5.0. 1.0 mL bacteria were added to a 2.0 mL Eppendorf tube and centrifuged at 12,000$\times$*g* for 5 min at $4\,^\circ$C. Supernatants were transferred to fresh 2 mL microcentrifuge tubes, and 600 $\upmu$L of phenol: chloroform: isoamyl alcohol (Sigma, Aldrich) with the ratio of 25: 24: 1, respectively, pH 6.7, was added for each extraction. Samples were incubated at room temperature for 10 min. Phase separation occurred during the centrifugation at 12,000$\times$*g* for 5 min at $4\,^\circ$C. Then, the upper aqueous phase was transferred to a new tube and added 450 $\upmu$L of isopropanol (Biobasic, Canada). The samples were incubated at $20\,^\circ$C overnight before being centrifuged at 12,000$\times$*g* for 5 min at $4\,^\circ$C, and supernatants were discarded. Finally, DNA pellets were washed in 1 mL of 70% (v/v) ethanol (Merck, Germany). The final pellet was dried in air and re-suspended in 100 $\upmu$L of 75 mM TE buffer pH 8.0. DNA was stored at $-\,20\,^\circ$C prior to use. All the DNA used in this study was measured the OD260/280, the results showed the ratios about 2.0. These indicators proved that the DNAs are pure.

### Absorbance measurements of DNA using UV–Vis method

In our experiments, 100 $\upmu$L of the probe and 1400 $\upmu$L of $\mathrm {MoS_2}$ with concentrations of 0.0625, 0.05, 0.04, 0.031, 0.025, 0.01, and 0.005 g/L were added to the curvet 10mm using TE buffer as solvent. These mixtures were ready to use as a sensing platform immediately. Then 100 $\upmu$L of DNA was repeatedly added to the cuvette to achieve different concentration levels (from 2 to 11.65 fM). At each level, the UV–Vis absorption spectrum was measured. In all our real-time experiments, the probe concentration was 35 nM. We evaluated the performance of seven sensors with various concentrations of the sensing material, to determine the optimal sensor configuration.

## Data Availability

The datasets used and analysed during the current study available from the corresponding author on reasonable request.

## References

[1] Kaper JB, Nataro JP, Mobley HL (2004). Pathogenic *Escherichia coli*. Nat. Rev. Microbiol..

[2] Ahmed A, Rushworth JV, Hirst NA, Millner PA (2014). Biosensors for whole-cell bacterial detection. Clin. Microbiol. Rev..

[3] Van de Beek D, de Gans J, Tunkel AR, Wijdicks EF (2006). Community-acquired bacterial meningitis in adults. N. Engl. J. Med..

[4] Law JW-F, Ab Mutalib N-S, Chan K-G, Lee L-H (2015). Rapid methods for the detection of foodborne bacterial pathogens: Principles, applications, advantages and limitations. Front. Microbiol..

[5] Li F (2010). Detection of *Escherichia coli* o157: H7 using gold nanoparticle labeling and inductively coupled plasma mass spectrometry. Anal. Chem..

[6] Yamada K (2014). Single walled carbon nanotube-based junction biosensor for detection of *Escherichia coli*. PLoS ONE.

[7] Chalklen T, Jing Q, Kar-Narayan S (2020). Biosensors based on mechanical and electrical detection techniques. Sensors.

[8] Zhou Y (2019). Electrochemical aptasensing strategy for kanamycin detection based on target-triggered single-strand dna adsorption on mos2 nanosheets and enzymatic signal amplification. Sens. Actuators B Chem..

[9] Dai Z, Hu X, Wu H, Zou X (2012). A label-free electrochemical assay for quantification of gene-specific methylation in a nucleic acid sequence. Chem. Commun..

[10] Oreshkin V, Tsizin G (2009). Atomic absorption determination of cadmium, lead, and mercury in sea and river suspensions using an electrothermal atomizer with two vaporization zones. J. Anal. Chem..

[11] Geng Y, Wu J, Shao L, Yan F, Ju H (2014). Sensitive colorimetric biosensing for methylation analysis of p16/cdkn2 promoter with hyperbranched rolling circle amplification. Biosens. Bioelectron..

[12] Singh P, Gupta R, Sinha M, Kumar R, Bhalla V (2016). Mos 2 based digital response platform for aptamer based fluorescent detection of pathogens. Microchim. Acta.

[13] Abu-Salah KM (2015). Dna-based nanobiosensors as an emerging platform for detection of disease. Sensors.

[14] Maki WC (2008). Nanowire-transistor based ultra-sensitive dna methylation detection. Biosens. Bioelectron..

[15] Voiry D (2013). Conducting mos2 nanosheets as catalysts for hydrogen evolution reaction. Nano Lett..

[16] Ma H, Shen Z, Ben S (2018). Understanding the exfoliation and dispersion of mos2 nanosheets in pure water. J. Colloid Interface Sci..

[17] Qu R (2018). A mos 2 nanosheet-coated mesh for ph-induced multi-pollutant water remediation with in situ electrocatalysis. J. Mater. Chem. A.

[18] Abinaya R (2018). Ultrathin layered mos 2 nanosheets with rich active sites for enhanced visible light photocatalytic activity. RSC Adv..

[19] Zhu C (2013). Single-layer mos2-based nanoprobes for homogeneous detection of biomolecules. J. Am. Chem. Soc..

[20] Xi Q (2014). Highly sensitive and selective strategy for microrna detection based on ws2 nanosheet mediated fluorescence quenching and duplex-specific nuclease signal amplification. Anal. Chem..

[21] Yang Y (2015). Mos2-based nanoprobes for detection of silver ions in aqueous solutions and bacteria. ACS Appl. Mater. Interfaces.

[22] Ramakrishna Matte H (2010). Mos2 and ws2 analogues of graphene. Angew. Chem. Int. Ed..

[23] Iwai H, Kakushima K, Wong H (2006). Challenges for future semiconductor manufacturing. Int. J. High Speed Electron. Syst..

[24] Sobańska Z, Zapor L, Szparaga M, Stępnik M (2020). Biological effects of molybdenum compounds in nanosized forms under in vitro and in vivo conditions. Int. J. Occup. Med. Environ. Health.

[25] Jagminas A (2019). Mos2 with organic fragment—A new hybrid material for laser writing. Sci. Rep..

[26] Liu H-Q, Yao C-B, Jiang C-H, Wang X (2021). Preparation, modification and nonlinear optical properties of semiconducting mos2 and mos2/zno composite film. Opt. Laser Technol..

[27] Cui S, Wen Z, Huang X, Chang J, Chen J (2015). Stabilizing mos2 nanosheets through sno2 nanocrystal decoration for high-performance gas sensing in air. Small.

[28] Liu L (2020). Edge-exposed mos2 nanospheres assembled with sns2 nanosheet to boost no2 gas sensing at room temperature. J. Hazard. Mater..

[29] Wang J (2021). Mos2-based nanocomposites for cancer diagnosis and therapy. Bioact. Mater..

[30] Tang, W. Electrical, electronic and optical properties of MoS2 & WS2. Master’s thesis, New Jersey Institute of Technology (2017).

[31] Karp M (1989). Expression of bacterial luciferase genes from *Vibrio harveyi* in bacillus subtilis and in *Escherichia coli*. Biochim. Biophys. Acta Gene Struct. Expr..

[32] Jin K, Xie L, Tian Y, Liu D (2016). Au-modified monolayer mos2 sensor for dna detection. J. Phys. Chem. C.

[33] Yan L, Shi H, Sui X, Deng Z, Gao L (2017). Mos 2-dna and mos 2 based sensors. RSC Adv..

[34] Faramarzi V, Ahmadi V, Fotouhi B, Abasifard M (2019). A potential sensing mechanism for dna nucleobases by optical properties of go and mos 2 nanopores. Sci. Rep..

[35] Jaiswal N, Pandey CM, Solanki S, Tiwari I, Malhotra BD (2020). An impedimetric biosensor based on electrophoretically assembled zno nanorods and carboxylated graphene nanoflakes on an indium tin oxide electrode for detection of the dna of *Escherichia coli* o157: H7. Microchim. Acta.

[36] Xiang X (2015). Mos2 nanosheet-based fluorescent biosensor for protein detection via terminal protection of small-molecule-linked dna and exonuclease iii-aided dna recycling amplification. Biosens. Bioelectron..

[37] Alexaki K (2021). A dna sensor based on upconversion nanoparticles and two-dimensional dichalcogenide materials. Front. Chem. Sci. Eng..

[38] Huang J (2015). Molybdenum disulfide-based amplified fluorescence dna detection using hybridization chain reactions. J. Mater. Chem. B.

[39] Nguyen SH, Vu PKT, Tran MT (2023). Glucose sensors based on chitosan capped Zns doped Mn nanomaterials. IEEE Sens. Lett..

